# Graves’ disease presenting as paranoid schizophrenia in a Nigerian woman: a case report

**DOI:** 10.4076/1757-1626-2-6708

**Published:** 2009-08-10

**Authors:** Okechukwu S Ogah, Anne O Timeyin, Oluyinka A Kayode, Adedeji S Otukoya, Rufus O Akinyemi, Folashade I Adeyemi

**Affiliations:** 1 Department of Medicine, Federal Medical CentrePMB 3031 Sapon, Abeokuta, Ogun StateNigeria; 2 Department of Psychiatry, Federal Medical CentrePMB 3031 Sapon, Abeokuta, Ogun StateNigeria

## Abstract

Paranoid syndromes in Graves’ disease are rare. The true incidence is lacking. Most reports have emanated from developed countries where medical investigations are readily available. No report of such has emanated from Nigeria. We report a 43-year-old female Nigerian with Graves’ disease associated with paranoid schizophrenia and review the literature.

## Introduction

More than a century ago, Basedow first described psychiatric illness (most probably mania) in a patient with Grave’s disease [1]. Since then several anecdotal case reports of psychiatric manifestations of hyperthyroidism have been reported [2-4]. However most medical textbooks do not mention psychosis as a presenting feature of hyperthyroidism. On the other hand psychosis associated with hypothyroidism is universally documented [5,6].

Paranoid syndromes in Graves’ disease are rare. The true incidence is lacking. Most reports have emanated from developed countries where medical investigations are readily available. To the best of our knowledge, the only attempt to report on psychiatric features of hyperthyroidism (in stable state) in Nigeria was by Kolawole *et al.* [7] where he reported two cases of mild depression out of the eight patients studied.

We report a 43-year-old female Nigerian with Graves’ disease associated with paranoid schizophrenia.

## Case presentation

A 43-year-old female Nigerian was referred to us from a nearby psychiatric hospital where she was hospitalized for four weeks on account of an eight months history of violent behavior, accusing her mother of being a witch, and 2 months history of keeping to self, poor sleep, talking and laughing to self, threatening to harm neighbours, poor personal hygiene and weight loss.

She was living in the UK prior to the onset of illness but often visited her relations in Nigeria from time to time. In November 2006, she had arrived in Nigeria unexpectedly, calling her mother from the airport and abusing her, calling her a witch. She accused her mother of stealing her property and that she had come to take them back. From then on, she was observed to be increasingly suspicious of her mother, and would not interact with other members of the family. She was also observed to be sleeping poorly, talking and laughing to self, and neglecting her personal hygiene. She moved out of the house to another apartment in the compound where she stayed alone and was often observed to be praying excessively. She accused her neighbours of wanting to harm her and then bought a cutlass, threatening to attack anyone who came near her. She resisted initial attempts to bring her to hospital.

There was no history of fever or head injury prior to onset of illness. No history of weepy spells, or feelings of worthlessness, hopelessness, loss of interest in previously enjoyable things, guilt feelings or suicidal ideation. No history of extravagant spending but at least on one occasion during this episode she was observed to be giving out some of her belongings without obvious reasons.

She was managed as paranoid schizophrenia and was placed on antipsychotic medications. Rather than improving, her condition worsened which necessitated a search for an organic cause of her illness. On discovery, that patient also had features of hyperthyroidism she was referred to us for medical treatment. She had noticed anterior neck swelling 30 months earlier in the UK and was placed on iodized salt by her general practitioner.

The neck swelling has been increasing in size. There was associated history of palpitations, excessive sweating, and tremulousness. Her friends had drawn her attention to protrusion of her eyes but can not recall when exactly. She also noticed increased pigmentation of her skin. There has been significant weight loss despite increased appetite. No associated passage of frequent loose stools. No history of change in the quality of the voice, difficulty in breathing or swallowing and neck pain.

Patient returned to Nigeria from the UK where she lived for 11 years about 8 months prior to presentation with the aim of setting up a small scale business. She had no past or family history of psychotic behavior. Prior to her return to Nigeria, she had worked as a social worker in the UK. She has no family history of psychiatric illness.

Clinical examination revealed a chronically ill - looking woman with staring gaze, lid lag, lid retraction and was mildly pale. She had tremors on the outstretched hand as well as increased pigmentation of the skin.

The anterior neck swelling, measured about 6 by 8 cm, moved with swallowing but not with protrusion of the tongue, firm to hard in consistency, with a smooth surface, no retrosternal extension present, no palpable regional lymph nodes present and no bruits were heard.

The central nervous system examination revealed a conscious agitated woman. Her speech was irrelevant, was deluded and had no insight.

The initial mental state examination showed that she was poorly groomed and restless. She was irritable and threatening as well as talkative and abusive. Her speech was irrational, with flight of ideas. There were persecutory delusions towards relatives, especially her mother. The patient was very uncooperative and had no insight.

She had a pulse of 120 beats/minute; blood pressure of 120/70 mmHg. The apex beat was not displaced and the heart sounds were normal. Chest and abdominal examination did not reveal any abnormality.

Thyroid function tests done before patient was referred to our centre showed: T - 330.9 ng/dL (Normal = 69-202 ng/dl), T4 -261.6 nmol/L (Normal -58.7-103 nmol/l) and TSH - 0.12 microIU/L (Normal = 0.32-5.2 microIU/L).

A repeat thyroid function carried out after weeks on antithyroid drugs revealed T3 - 2.1 ng/ml (0.8-2.0), T4 - 163 ng/ml (45-115), TSH - 0.1 microIU/ml (0.54-3.7) and anti thyroid antibody value - 69.5 IU/mL (3.5-7.6). Thyroid ultrasound scan done showed a multinodular goitre. Patient had a Packed Cell Volume of 26%; Total white cell count was 3.5 × 10/L^9^, (differentials - Neutrophils 30%, lymphocytes 66%, monocytes 4%, basophils 0%, eosinophils 0%). Platelet count was 160,000/L. Blood film showed the presence of hypochromic erythrocytes, relative lymphocytosis. Erythrocyte sedimentation rate was 28 mm/hr (westergreen).

Patient was admitted and placed on oral propranolol 40 mg thrice daily, oral carbimazole 10 mg thrice daily, oral Chlorpromazine 200 mg twice daily and oral artane 5 mg daily.

She was initially placed on oral Haloperidol 15 mg daily in divided doses, with intramuscular chlorpromazine given prn for severe agitation at the psychiatric hospital. When there was no significant reduction in her level of agitation after a week on these medications, haloperidol was discontinued and oral chlorpromazine commenced.

During the first few days on admission, she was aggressive, restless, hallucinated and sometimes trying to attack other patients which necessitated four point restrain for few days. Additional therapy includes oral dexamethsone 4 mg twice daily.

Over the next few days she became calm, well oriented but exhibited swinging mood (but in most of the time showed features of mania) and was gradually weaned off anti-psychotics.

She responded well to anti-thyroid drugs. Within eight weeks she became euthyroid. On follow-up (over 12 months now), she has remained symptom free and has since returned to her work and there has not been recurrence of psychiatric symptoms.

## Discussion

The case presented is remarkable in that psychotic symptoms are uncommon in Graves’ disease and usually occur in the thyrotoxic state [1,8]. Even in this situation, the association of paranoid schizophrenia is still controversial [9].

Common psychiatric syndromes in hyperthyroidism are anxiety and mood disturbances. The thyroid hormone is said to influence the functioning of the brain and can interact with mood regulation through targets in specific brain connections [10,11]. The paranoid schizophrenia in this case must have been due to thyrotoxicosis since it could not be attributed to any other organic conditions in the patient. More so her clinical symptoms did not improve with anti-psychotic therapy she received for 4-weeks at the psychiatric hospital. There was also no history of ingestion of exogenous thyroid preparations although facilities were not available for Technecium-99 m pyrophosphate thyroid scan.

She was offered subtotal thyroidectomy but she declined. Complete resolution of psychosis has been reported with any of these treatments supporting the assumption that the causal relationship between Graves’ disease and organic psychosis [8]. The mechanism for this is still unclear. One school of thought believes that fall in anti-thyroid antibody may be responsible.

## Conclusion

Hyperthyroidism can present with many psychiatric syndromes including paranoid schizophrenia. A high sense of suspicion is often required to detect the association.
